# Cytoreductive nephrectomy with thrombectomy before targeted therapy improves survival for metastatic renal cell carcinoma with venous tumor thrombus: a single-center experience

**DOI:** 10.1186/s12957-016-1066-3

**Published:** 2017-01-05

**Authors:** Nienie Qi, Pengjie Wu, Jinchao Chen, Teng Li, Xianghui Ning, Jin Wang, Kan Gong

**Affiliations:** 1 Department of Urology, Peking University First Hospital, Institute of Urology, Peking University, Beijing, China; 2 Department of Urology, Beijing Hospital, Beijing, China; 3 Department of cardiac surgery, Peking University First Hospital, Beijing, China; 4 Department of Urology, Peking University First Hospital, Institute of Urology, Peking University; National Urological Cancer Center, No. 8, Xishiku St., Xicheng Dist, Beijing, 100034 China

**Keywords:** Renal cell carcinoma, Metastasis, Venous tumor thrombus, Cytoreductive surgery, Targeted molecular therapy

## Abstract

**Background:**

The aim of the study is to evaluate the role of cytoreductive nephrectomy (CN) with thrombectomy before targeted molecular therapy (TMT) on survival in metastatic renal cell carcinoma (mRCC) with venous tumor thrombus.

**Methods:**

We performed a retrospective analysis of 47 patients treated in our center from April 2008 to October 2014. In the study, 20 patients underwent CN with thrombectomy followed by targeted therapy (group 1); 15 patients received targeted therapy alone (group 2); and 12 patients underwent CN with thrombectomy alone (group 3). The overall survival (OS) and cancer-specific survival (CSS) were calculated according to the Kaplan-Meier survival curve method, and prognostic variables were assessed by Cox regression analyses.

**Results:**

The median follow-up times of group 1, group 2, and group 3 were 24.5, 12, and 6.5 months, respectively. During follow-up, in both group 1 and group 3, 12 patients died. In group 2, 14 patients died. The median OS of group 1, group 2, and group 3 was 22, 12, and 6 months, respectively (*P* < 0.001). Compared with surgery alone and targeted therapy alone, patients with cytoreductive surgery before targeted therapy had statistically better survival benefits (*P* < 0.001, *P* = 0.009, respectively). On univariate analysis, the number of metastatic sites (*P* = 0.004) was a statistically significant prognostic factor influencing OS.

**Conclusions:**

Our single-center experience showed that CN with thrombectomy before targeted therapy improved the survival of patients with mRCC with venous tumor thrombus. The number of metastatic sites was an independent prognostic factor influencing OS.

## Background

Venous tumor thrombus (VTT) occurs in approximately 5–10% of patients undergoing nephrectomy for renal cell carcinoma (RCC) [1]. In addition to venous involvement, 25% of patients with RCC have metastatic disease when diagnosed [2]. Patients with RCC and VTT should be considered for surgical intervention, irrespective of the extent of tumor thrombus at presentation [3]. Before the era of targeted therapy, two prospective randomized controlled trials had demonstrated the overall benefit of cytoreductive nephrectomy (CN) in metastatic RCC [4, 5]. A population-based study also showed the beneficial effect of CN on survival [6]. The emerging of targeted therapy offers more choices for mRCC and has prompted a reevaluation of cytoreductive surgery [7]. Tyrosine kinase inhibitor (TKI) had been shown to extend the progression-free survival and overall survival [8]. Differential expression of prognostic proteomic markers, such as VEGFR1, was found in primary tumor, VTT, and metastatic renal cell cancer tissue [9], suggesting that TKI alone may help improve the survival of mRCC with VTT.

Although CN with inferior vena cava (IVC) thrombectomy was performed with acceptable complication rates [10], it remains unclear whether surgery is still a good choice for concomitant mRCC with VTT. Thus, we retrospectively evaluated the benefits of CN with thrombectomy before targeted therapy in patients presenting with mRCC with VTT.

## Methods

We reviewed the medical records of all patients who presented to our center from April 2008 to October 2014 for evaluation of or treatment for concomitant mRCC with VTT. During the study period, 61 patients presented with concomitant mRCC with VTT who had received no systemic therapy. Among them, 20 patients underwent CN with thrombectomy followed by TMT (combined therapy, group 1); 15 patients received TMT alone (group 2); 12 patients underwent CN with thrombectomy alone (group 3); and 2 refused any therapy. The treatment of the other 12 patients was uncertain. Patients in group 1, group 2, and group 3 had postoperative pathology or biopsy confirmed of RCC histology. Metastasis was confirmed by radiology. The following clinical characteristics of each patient were recorded: age, gender, ECOG PS (Eastern Cooperative Oncology Group performance score), tumor size, pathologic type, nuclear grade, venous thrombus level, number of metastatic sites, T stage and N stage. These characteristics were potential prognostic factors [11–14]. Staging was determined according to the 2009 AJCC staging system [15]. Nuclear grade was graded using the Fuhrman grading system [16]. Venous tumor extent was graded according to the Mayo Clinic grading system [17]. Nineteen patients received sorafenib (18 cases as first-line therapy and 1 case as second-line therapy following sunitinib). Sorafenib was administered at a dose of 400 mg twice daily and continued until disease progression or the onset of an intolerable adverse drug event. The 19 patients received sunitinib (16 cases as first-line therapy and 3 cases as second-line therapy following sorafenib). Sunitinib was administered at a dose of 50 mg once daily for 4 weeks, followed by 2 weeks off by repeated 6-week cycles. All the 19 patients were treated continuously until disease progression or unacceptable toxicities occurred. Pazopanib monotherapy was administered at a dose of 800 mg once daily in 1 patient. No neoadjuvant targeted therapy was used. Informed consent was obtained from all individual participants included in the study. The study was approved by the institutional review board from Peking University First Hospital.

### Statistical analysis

Likelihood ratio with chi-square and *t* tests was used for comparisons between groups in categorical and continuous variables, respectively. OS (overall survival) and CSS (cancer-specific survival) curves were derived by the Kaplan-Meier method with the log-rank test. Each group was further compared with Kaplan-Meier curve. Univariate Cox hazards regression were applied to evaluate the value of prognostic factors including advanced age, gender, ECOG PS, pathological type, nuclear grade, venous thrombus level, T stage, N stage, and number of metastatic sites in predicting OS and CSS. All statistical analyses were performed using SPSS version 19 (SPSS Inc., Chicago, IL, USA). Statistical significance was set at *P* < 0.05.

## Results

Table 1 shows patient clinicopathological characteristics at diagnosis. The median age of group 1, group 2, and group 3 were 55(20–70), 58(26–72), and 61(46–71) years, respectively. There were no significant differences except gender, ECOG PS, nuclear grade, N stage, and number of metastatic sites. In group 1, 10 patients were T3a; 5 patients were T3b. The number of level 0, level II, and level III thrombus was 12, 5, and 3, respectively. Lung metastasis, liver metastasis, bone metastasis, and adrenal metastasis appeared in 12, 4, 3, and 2 patients, respectively. In group 2, 4 patients were T3a; 8 patients were T3b. The number of level 0, level I, level II, and level III thrombus was 4, 1, 3 and 7, respectively. Lung metastasis, liver metastasis, adrenal metastasis, bone metastasis, and pleura metastasis appeared in 10, 5, 4, 3, and 2 patients, respectively. In group 3, 7 patients were T3a; 1 patient was T3b; 1 patient was T3c. The number of level 0, level I, level III, and level IV thrombus was 9, 1, 1 and 1, respectively. Lung metastasis, bone metastasis, liver metastasis, and adrenal metastasis appeared in 9, 3, 1, and 1 patient, respectively.

**Table 1:** Patient demographics of group 1, group 2, and group 3

	Group 1	Group 2	Group 3	*P*
No	20	15	12	
Gender, *n* (%)				0.590^a^,0.022^b^
Male	15(75.0%)	10(66.7%)	12(100%)	
Female	5(25.0%)	5(33.3%)	0(0%)	
Age, *n* (%)				0.486^a^,0.197^b^
≥60	7(35.0%)	7(46.7%)	7(58.3%)	
<60	13(65.0%)	8(53.3%)	5(41.7%)	
Pathologic type, *n* (%)				1.000^a^,0.360^b^
Clear cell	16(80.0%)	12(80.0%)	11(91.7%)	
Non-clear cell	4(20.0%)	3(20.0%)	1(8.3%)	
Nuclear grade, *n* (%)				0.005^a^,0.488^b^
G1 + G2	3(15.0)	9(60.0%)	3(25.0%)	
G3 + G4	17(85.0%)	6(40.0%)	9(75.0%)	
VT level, *n* (%)				0.039^a^,0.900^b^
Above hepatic vein	3(15.0%)	7(46.7%)	2(16.7%)	
Below hepatic vein	17(85.0%)	8(53.3%)	10(83.3%)	
Tumor size, (cm ± SD)	9.9 ± 2.4	10.8 ± 4.0	9.8 ± 1.8	0.439^a^,0.903^b^
T stage, *n* (%)				0.726^a^,1.000^b^
T3	15(75.0%)	12(80.0%)	9(75.0%)	
T4	5(25.0%)	3(20.0%)	3(25.0%)	
N stage, *n* (%)				0.061^a^,0.252^b^
N0	13(65.0%)	5(33.3%)	10(83.3%)	
N1	7(35.0%)	10(66.7%)	2(16.7%)	
Number of metastatic sites, *n* (%)				0.001^a^,0.586^b^
1	18(90.0%)	6(40.0%)	10(83.3%)	
>1	2(10.0%)	9(60.0%)	2(16.7%)	
ECOG PS				0.015^a^,0.742^b^
0	16(75.0%)	6(40.0%)	9(75.0%)	
1	4(25.0%)	9(60.0%)	3(25.0%)	

^a^Comparison between group 1 and group 2

^b^Comparison between group 1 and group 3

**Fig. 1:**
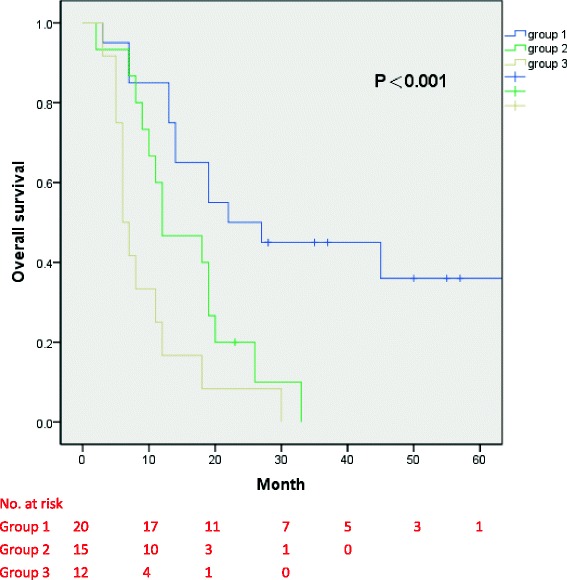
Overall survival of group 1, group 2, and group 3

**Fig. 2:**
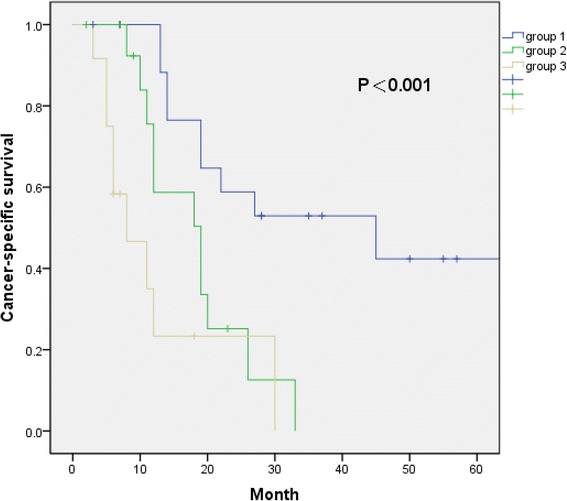
Cancer-specific survival of group 1, group 2, and group 3

The median follow-up time of group 1, group 2, and group were 24.5 (range 3–66), 12 (range 2–33), and 6.5 (range 3–30) months. During follow-up, 12 patients died in both group 1 and group 3. Fourteen patients died in group 2. The median OS of group 1, group 2, and group 3 was 22 (95% CIs 4.5–39.5), 12 (95% CIs 3.2–20.8), and 6 (95% CIs 4.3–7.7) months, respectively (*P* < 0.001, Fig. 1). The median CSS of group 1, group 2, and group 3 was 45, 19, and 8 months, respectively (*P* < 0.001, Fig. 2). The median progression-free survival of group 1 and group 2 were 12 (range 1–66) and 9 (range 2–23) months, respectively. Compared with patients who underwent surgery alone or targeted therapy alone, patients with cytoreductive surgery before targeted therapy had statistically better overall survival benefits (*P* < 0.001 and *P* = 0.009, respectively). Patients with combined therapy also had better CSS benefits. In a univariate Cox proportional hazards model to predict OS, we found that the number of metastatic sites was a significant predictor of OS (Table 2). Compared with patients with solitary metastasis, patients with more than one metastatic site had a 2.94-fold higher risk of overall mortality (95% CIs 1.41–6.09, *P* = 0.004). However, the number of metastasis did not affect the probability of CSS (Table 2).

**Table 2:** Univariate analysis of potential prognostic factors for OS and CSS

	Univariate for OS		Univariate for CSS	
Variables	HR(95% CI)	*P* value	HR(95% CI)	*P* value
Age (≥60 vs. <60)	1.56(0.82–2.96)	0.178	1.53(0.73–3.21)	0.256
Gender (female vs. male)	1.02(0.48–2.17)	0.958	1.07(0.45–2.51)	0.883
ECOG PS (1 vs. 0)	0.59(0.29–1.20)	0.145	0.64(0.29–1.41)	0.267
Pathological type (non-clear cell vs. clear cell)	0.71(0.28–1.83)	0.480	0.76(0.26–2.18)	0.605
Nuclear grade (G3 + G4 vs. G1 + G2)	0.84(0.43–1.64)	0.608	0.65(0.31–1.40)	0.273
VT level (above hepatic vein vs. below hepatic vein)	1.67(0.71–3.03)	0.302	1.36(0.58–3.20)	0.482
T stage (T4 vs. T3)	1.50(0.70–3.20)	0.294	1.63(0.69–3.87)	0.264
N stage (N1 vs. N0)	1.32(0.69–2.54)	0.407	1.63(0.77–3.47)	0.203
Number of metastatic sites (more than 1 vs. 1)	2.94(1.41–6.09)	0.004	2.14(0.86–5.34)	0.103
Combined therapy				
TMT alone	2.69(1.20–6.02)	0.017	3.25(1.28–8.27)	0.013
Surgery alone	5.64(2.39–13.29)	<0.001	7.05(2.60–19.11)	<0.001

One patient in group 1 achieved complete remission. None of patients in group 2 achieved complete remission. The median treatment duration of sorafenib was 8 months. Adverse events were shown in Table 3. Most of them were grade 1–2 adverse events. The grade 3–4 major adverse events (4 cases) included hand-foot syndrome (2 cases), rash (1 case), and fatigue (1 case). All of them kept treatment with symptomatic support. The median treatment duration of sunitinib was 12 months. Adverse events were shown in Table 4. The grade 3–4 major adverse events (7 cases) included thrombocytopenia (3 cases), leukocytopenia (2 cases), hand-foot syndrome (1 case), and thyroid dysfunction (1 case). Six patients had dose decrement or drug discontinuation. All of them kept treatment with symptomatic support. The patient treated with pazopanib emerged with hand-foot syndrome, nausea, and vomit during treatment. After 16 months, he progressed with lung metastasis and withdrew from treatment.

**Table 3:** Adverse events of sorafenib

Adverse events	Grade 1–2, *n*	Grade 3–4, *n*	*n*, %
Diarrhea	11	0	11(57.9%)
Hand-foot syndrome	9	2	11(57.9%)
Hypertension	8	0	8(42.1%)
Alopecia	7	0	7(36.8%)
Rash	5	1	6(31.6%)
Fatigue	1	1	2(10.5%)
Nausea and vomit	1	0	1(5.3%)
Anemia	1	0	1(5.3%)
Leukocytopenia	1	0	1(5.3%)
Liver dysfunction	1	0	1(5.3%)
Elevation of uric acid	1	0	1(5.3%)

**Table 4:** Adverse events of sunitinib

Adverse events	Grade 1–2, *n*	Grade 3–4, *n*	*n*, %
Thrombocytopenia	12	3	15(78.9%)
Thyroid dysfunction	13	1	14(73.7%)
Hand-foot syndrome	12	1	13(68.4%)
Leukocytopenia	9	2	11(57.9%)
Hypertension	9	0	9(47.4%)
Fatigue	8	0	8(42.1%)
Diarrhea	8	0	8(42.1%)
Anorexia	7	0	7(36.8%)
Palpebral edema	6	0	6(31.6%)
Oral mucositis	5	0	5(26.3%)
Hypophosphatemia	3	0	3(15.8%)
Liver dysfunction	1	0	1(5.3%)

## Discussion

The combination of CN and systemic therapy plays an important role in the management of patients with mRCC. Retrospective studies suggest that mRCC patients with IVC tumor thrombus may experience improved survival after surgical resection and systemic therapy. These studies were conducted before the current era of targeted therapy, which, at present, represent a standard therapy for mRCC. In the era of targeted therapy, few researches explored this problem. Taekmin Kwon et al. found that surgical resection of the primary renal mass with IVC thrombus before use of TKI did not affect the overall mortality [18]. Conversely, Karin E et al. suggested that CN with IVC thrombectomy should be considered as an integral part of the treatment approach for patients with mRCC with IVC tumor thrombi [10]. However, proper treatment of mRCC with VTT has yet to be determined. Our study showed that compared with surgery alone and targeted therapy alone, cytoreductive surgery before targeted therapy improved the overall survival and cancer-specific survival of mRCC with VTT.

The prognosis was quite poor for the majority of patients with RCC with VTT when left untreated, especially for those with metastases. In patients presenting with metastatic disease, 1-year DSS (disease-specific survival) was 23% (median DSS: 4 months) for those with thrombus below the diaphragm and 10% (median DSS: 3 months) for those with thrombus above the diaphragm [19]. For those with distant metastases at the time of diagnosis who underwent surgery, 1-year survival was 60%. This finding highlighted the importance of surgery on their prognosis. With the development of surgical skills, novel anesthetic technique, and hemodynamic monitor, the perioperative mortality and complications associated with nephrectomy with thrombectomy decreased to an acceptable level [17, 20]. In some patients, surgery may help to relieve symptoms. However, the surgery was still challenging, and surgery-related morbidity or mortality could not be neglected. Bissada et al. reported higher perioperative mortality in patients with metastasis (33%) versus patients without metastasis (2%) [21]. Fortunately, all of the 32 patients survived surgery in our center. However, some patients may undergo disease progression during recovery from surgery and may not proceed to receive systemic therapy for their metastatic tumor burden [22]. In fact, we observed that some patients failed to receive targeted therapy after surgery for several reasons. Silberstein et al. [23] have demonstrated that patients with poor performance status are more likely to experience postoperative complications, and these patients tend not to receive systemic therapy on time after surgery. Culp et al. [24] identified seven risk factors that predicted inferior OS after CN, including high lactate dehydrogenase activity, low albumin level, symptoms caused by metastatic disease, liver metastasis, retroperitoneal adenopathy, supradiaphragmatic adenopathy, and a greater than cT3 disease. Patients with ≥4 risk factors did not appear to benefit from CN. Based on the present study, it seems that patients’ general condition and performance status should be used to best define candidates for CN with VTT. While the risk of perioperative complications outweighed the potential benefits, cytoreductive surgery should not be the first option considered.

On univariate Cox regression analysis, pathologic type, nuclear grade, and venous tumor thrombus level were not significantly associated with survival. In contrast, more than one metastatic site was an independent prognostic factor of a poor OS. In fact, a previous study had already shown that the number of metastatic sites affected the survival rates of the patients with mRCC [25]. This may be owing to the more disease burden and worse functional status. More studies are needed to help ameliorate the poor prognosis.

Our study had some limitations. The number of patients was low and follow-up time was short. Because of its retrospective nature, the study lacked randomization and has selection bias. Many factors may influence the decision to undergo cytoreductive surgery before targeted therapy. One obvious bias is that patients choosing targeted therapy alone have a higher percentage of multiple metastatic sites. Pierorazio PM has already shown that patients with widespread metastatic burden may not benefit from surgery and are likely to choose conservative management [26]. A population-based study based on the surveillance, epidemiology, and end results database revealed a similar result [27]. Some patients even failed to receive targeted therapy after surgery for various reasons. In additional, these patients had not been resected of metastatic sites, even though complete metastasectomy provides benefits in terms of OS, CSS, and delay of systemic therapy [3]. Moreover, in our study, the population was small, so we did not introduce multivariable Cox analysis. Large-scale, randomized, and prospective studies are needed to determine the effect on survival of CN with thrombectomy before targeted therapy.

## Conclusions

Compared with cytoreductive surgery alone and targeted therapy alone, CN with thrombectomy before targeted therapy may offer better survival for mRCC with VTT. Our single-center experience suggested a positive role for cytoreductive surgery followed by targeted therapy in these patients.

## Abbreviations

CN: Cytoreductive nephrectomy
CSS: Cancer-specific survival
DSS: Disease-specific survival
ECOG PS: Eastern Cooperative Oncology Group performance score
IVC: Inferior vena cava
mRCC: Metastatic renal cell carcinoma
OS: Overall survival
TKI: Tyrosine kinase inhibitor
TMT: Targeted molecular therapy
VTT: Venous tumor thrombus
